# The crystal structures of two novel polymorphs of bis­(oxonium) ethane-1,2-di­sulfonate

**DOI:** 10.1107/S2056989019013367

**Published:** 2019-10-03

**Authors:** Jaroslaw Mazurek, Ana Fernandez-Casares

**Affiliations:** a Ardena, Solid State Research Services, Meibergdreef 31, 1105 AZ Amsterdam, The Netherlands

**Keywords:** crystal structure, polymorphism, oxonium cation, sulfonate anions

## Abstract

Two novel crystal forms of bis­(oxonium) ethane-1,2-di­sulfonate, 2H_3_O^−^·C_2_H_4_O_6_S_2_^2−^, are reported. Polymorph II has monoclinic (*P*2_1_/*n*) symmetry, while the symmetry of form III is triclinic (*P* ). Both structures display extensive networks of O—H⋯O hydrogen bonds. While this network in Form II is similar to that observed for the previously reported Form I [Mootz & Wunderlich (1970 ▸). *Acta Cryst.* B**26**, 1820–1825; Sartori *et al.* (1994 ▸). *Z. Naturforsch.* **49**, 1467–1472] and extends in all directions, in Form III it differs significantly, forming layers parallel to the *ab* plane. The sulfonate mol­ecule in all three forms adopts a nearly identical geometry. The other observed differences between the forms, apart from the hydrogen-bonding network, are observed in the crystal density and packing index.

## Abstract 2

Two novel crystal forms of bis­(oxonium) ethane-1,2-di­sulfonate, 2H_3_O^−^·C_2_H_4_O_6_S_2_^2−^, are reported. Polymorph II has monoclinic (*P*2_1_/*n*) symmetry, while the symmetry of form III is triclinic (*P* 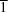). Both structures display extensive networks of O—H⋯O hydrogen bonds.

## Chemical context   

Sulfonic acids are commonly used in salt formation in the pharmaceutical industry, especially for poorly or non soluble in water drugs (Neau & Loka, 2018 ▸). Salts of ethane-1,2-di­sulfonic acid account for 0.38% of all the FDA-approved commercially marketed salts (Steele & Talbir, 2016 ▸) and therefore its toxicology, dosage (Saal & Becker, 2013 ▸) and various physico-chemical properties are widely studied (Black *et al.*, 2007 ▸; Elder *et al.*, 2010 ▸). In our laboratory, ethane-1,2-di­sulfonic acid is commonly used in the salt screening for increasing solubility as well as improving the crystallinity of various researched active pharmaceutical ingredients (APIs).
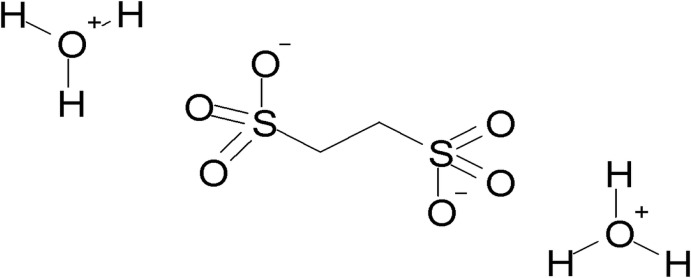


## Structural commentary   

**Figure 1:**
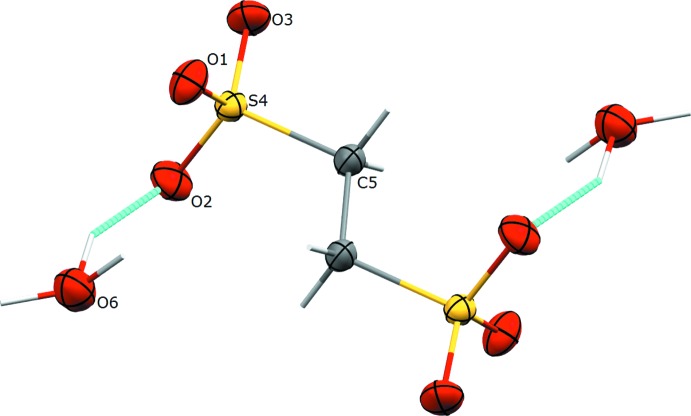
The mol­ecular structure of an anion–cation pair of Form II, with the atom-labelling scheme. Displacement ellipsoids are drawn at the 50% probability level and hydrogen bonds are shown in torquoise. Unlabelled atoms are related to labelled ones by the symmetry operator (−*x* + 1, −*y* + 1, −*z* + 1).

**Figure 2:**
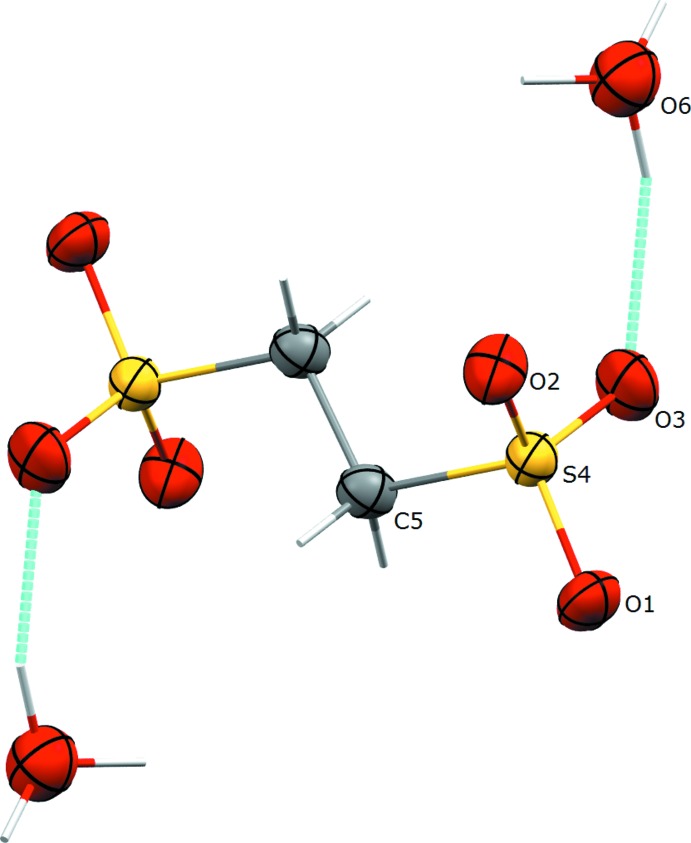
The mol­ecular structure of anion cation pair of Form III, with the atom-labelling scheme. Displacement ellipsoids are drawn at the 50% probability level and hydrogen bonds are shown in torquoise. Unlabelled atoms are related to labelled ones by the symmetry operator (−*x* + 1, −*y* + 1, −*z* + 1).

The sulfonate anion in all polymorphs, including the previously determined form (Mootz & Wunderlich, 1970 ▸, refcode HOEDSO; Sartori *et al.*, 1994 ▸, refcode HOEDSO01) has a nearly identical geometry. In all cases, the center of the C—C bond is located on an inversion center, and the C—S and C—O distances in all cases are within 3σ. The sulfonate group adopts the geometry of an open umbrella with the C—S—O bond angles of 106.51 (6), 105.82 (6), 107.23 (6)° for Form II (Fig. 1 ▸) and 106.16 (11), 106.21 (10), 107.20 (12)° for Form III (Fig. 2 ▸). The values of all O—S—O angles are above 110° [112.91 (7), 111.48 (7), 112.37 (7)° for Form II and 111.31 (11), 113.45 (11), 112.00 (12)° for Form III]. In this way, the mol­ecular symmetry of the sulfonate group becomes slightly distorted *C*_3*V*_. In all crystals, the oxonium cations have a pyramidal geometry with slightly elongated O—H distances for one H atom. This is most likely an effect of the fast exchange of a proton (H atom) between the sulfonate group and the water mol­ecules.

The biggest differences between forms are observed in the density of the crystal, as well as in the packing coefficient (Kitajgorodskij, 1973 ▸). The lowest values of both parameters are attributed to Form III (1.60 g cm^−3^ and 0.67, respectively), which suggests that this polymorph is the least stable. Form II presented here has a slightly better packing index than previously reported for Form I (Mootz & Wunderlich, 1970 ▸; Sartori *et al.*, 1994 ▸) 0.75 *versus* 0.73. On the other hand, the density is lower: 1.78 *versus* 1.82 g cm^−3^, respectively.

## Supra­molecular features   

**Figure 3:**
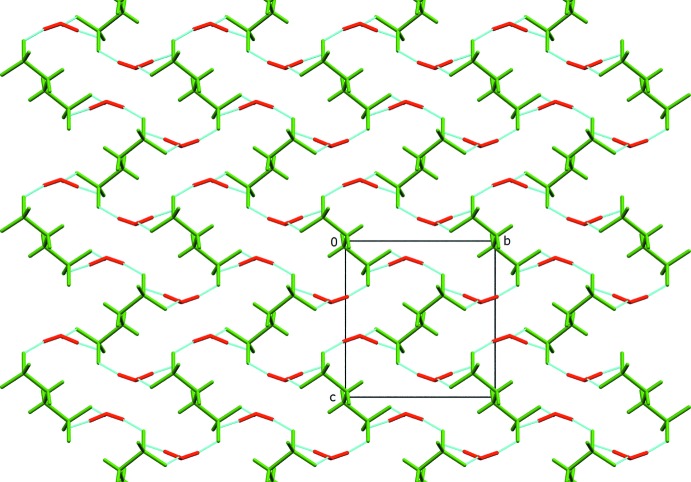
The crystal packing of Form II, viewed along the *a* axis. The ethane-1,2-di­sulfonate dianions are coloured in green, while oxonium cations are red and hydrogen bonds are shown in torquoise.

**Figure 4:**
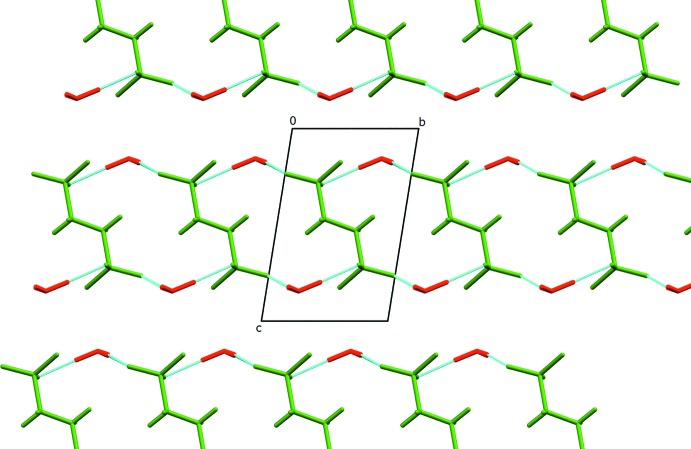
The crystal packing of Form III, viewed along the *a* axis. The ethane-1,2-di­sulfonate dianions are coloured in green, while oxonium cations are red and hydrogen bonds are shown in turquoise.

The hydrogen bonds between the oxonium cations and sulfonate anions in the crystal of Form II (Table 1 ▸, Fig. 3 ▸) extend in all directions forming a three-dimensional network similar to that observed for Form I (Mootz & Wunderlich, 1970 ▸; Sartori *et al.*, 1994 ▸). However, contrary to the previously reported form, where the hydrogen-bond network is built from alternate anion–cations layers, in Form II such layers could not be distinguished. The supra­molecular behaviour of Form III is significantly different. In this case (Table 2 ▸ and Fig. 4 ▸), the anion–cation hydrogen-bond network forms separate layers parallel to the *ab* plane built from sulfonate anions surrounded by oxonium cations with no inter­actions between the planes.

## Database survey   

As mentioned above, the crystal structure of a different polymorphic form of oxonium ethane-1,2-di­sulfonate has been previously reported (Mootz, & Wunderlich, 1970 ▸, refcode HOEDSO; Sartori *et al.*, 1994 ▸, refcode HOEDSO01). Apart from these structures, there are 12 hits for ethane-1,2-di­sulfonate salts in the Cambridge Structural Database (CSD, Version 5.40; *ConQuest 2.02*; Groom *et al.*, 2016 ▸), one of which is disordered. The geometry of the sulfonate group in all of the anions is nearly the same, with slightly distorted *C*_3*v*_ mol­ecular symmetry for the open-umbrella geometry. The average values of the C—S—O and O—S—O bond angles are very close to those reported in this paper: 105.9±0.8 and 112.8±0.9°, respectively.

## Synthesis and crystallization   

Both crystals were obtained from an aqueous solution during unsuccessful salt formation with an unnamed free base (API) in water. Firstly, columnar crystals of Form III that appeared to be unstable were grown from the thick oil and within time transformed into prismatic crystals of Form II.

## Refinement   

Crystal data, data collection and structure refinement details are summarized in Table 3 ▸. All H atoms were found in difference-Fourier maps and refined with isotropic displacement parameters. The DFIX 0.98 0.03 O6 H61, O6 H62 and O6 H63 instruction in *SHELXL2014/7* (Sheldrick, 2015*b* ▸) was used to restrain the oxonium O—H distance in Form II. All of the oxonium H atoms in Form III were refined independently without any restraints.

## Supplementary Material

Crystal structure: contains datablock(s) I, II. DOI: 10.1107/S2056989019013367/lh5920sup1.cif

Structure factors: contains datablock(s) I. DOI: 10.1107/S2056989019013367/lh5920Isup2.hkl

Supporting information file. DOI: 10.1107/S2056989019013367/lh5920Isup4.cml

Structure factors: contains datablock(s) II. DOI: 10.1107/S2056989019013367/lh5920IIsup3.hkl

Supporting information file. DOI: 10.1107/S2056989019013367/lh5920IIsup5.cml

CCDC references: 1956689, 1956690, 1956689, 1956690

Additional supporting information:  crystallographic information; 3D view; checkCIF report

## Figures and Tables

**Table 1:** Hydrogen-bond geometry (Å, °) for Form II[Chem scheme1]

*D*—H⋯*A*	*D*—H	H⋯*A*	*D*⋯*A*	*D*—H⋯*A*
O6—H63⋯O2	0.99 (2)	2.62 (2)	3.1795 (17)	116 (2)
O6—H61⋯O2^i^	1.00 (2)	2.02 (3)	2.9312 (16)	150 (3)
O6—H62⋯O3	1.06 (2)	1.92 (3)	2.9141 (16)	154 (3)
O6—H61⋯O3^ii^	1.00 (2)	2.60 (3)	2.9857 (16)	103 (2)
O6—H63⋯O1^iii^	0.99 (2)	2.14 (2)	3.0266 (18)	148 (2)

Symmetry codes: (i) 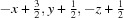; (ii) 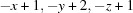; (iii) 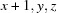.

**Table 2:** Hydrogen-bond geometry (Å, °) for Form III[Chem scheme1]

*D*—H⋯*A*	*D*—H	H⋯*A*	*D*⋯*A*	*D*—H⋯*A*
O6—H61⋯O1^i^	1.07 (4)	1.93 (4)	2.991 (3)	170 (4)
O6—H62⋯O2^ii^	1.02 (4)	2.52 (3)	3.002 (3)	108 (2)
O6—H62⋯O3	1.02 (4)	1.97 (4)	2.945 (3)	158 (3)
O6—H63⋯O1^iii^	1.02 (4)	1.89 (4)	2.899 (3)	173 (3)

Symmetry codes: (i) 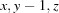; (ii) 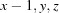; (iii) 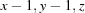.

**Table 3:** Experimental details

	Form II	Form III
Crystal data		
Chemical formula	2H_3_O^+^·C_2_H_4_O_6_S_2_^2−^	2H_3_O^+^·C_2_H_4_O_6_S_2_^2−^
*M*_r_	226.22	226.22
Crystal system, space group	Monoclinic, *P*2_1_/*n*	Triclinic, *P* 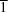
Temperature (K)	296	296
*a*, *b*, *c* (Å)	5.8050 (3), 8.3566 (6), 8.7433 (6)	5.0371 (3), 5.5424 (2), 8.8188 (4)
α, β, γ (°)	90, 95.148 (4), 90	98.426 (5), 104.511 (3), 91.663 (4)
*V* (Å^3^)	422.43 (5)	235.22 (2)
*Z*	2	1
Radiation type	Mo *K*α	Mo *K*α
μ (mm^−1^)	0.64	0.58
Crystal size (mm)	0.45 × 0.32 × 0.23	0.30 × 0.12 × 0.11

Data collection		
Diffractometer	Bruker KappaCCD	Bruker KappaCCD
Absorption correction	Gaussian integration (Coppens, 1970 ▸)	Gaussian integration (Coppens, 1970 ▸)
*T*_min_, *T*_max_	0.748, 0.907	0.813, 0.947
No. of measured, independent and observed [*I* > 2σ(*I*)] reflections	17906, 1848, 1768	7504, 1708, 1192
*R*_int_	0.075	0.131
(sin θ/λ)_max_ (Å^−1^)	0.806	0.758

Refinement		
*R*[*F* ^2^ > 2σ(*F* ^2^)], *wR*(*F* ^2^), *S*	0.041, 0.121, 1.04	0.058, 0.163, 1.04
No. of reflections	1848	1708
No. of parameters	76	76
No. of restraints	3	0
H-atom treatment	All H-atom parameters refined	All H-atom parameters refined
Δρ_max_, Δρ_min_ (e Å^−3^)	0.62, −0.93	0.66, −0.67

Computer programs: *COLLECT* (Hooft, 1998 ▸), *HKL* *SCALEPACK* and *DENZO* (Otwinowski & Minor, 1997 ▸), *SHELXT* (Sheldrick, 2015*a* ▸), *SHELXL2014/7* (Sheldrick, 2015*b* ▸), *Mercury* (Macrae *et al.*, 2008 ▸) and *enCIFer* (Allen *et al.*, 2004 ▸).

## supplementary crystallographic information

### Bis(oxonium) ethane-1,2-disulfonate (I) Crystal data

**Table:**

2H_3_O^+^·C_2_H_4_O_6_S_2_^2^^−^	*F*(000) = 236
*M**_r_* = 226.22	*D*_x_ = 1.778 Mg m^−^^3^
Monoclinic, *P*2_1_/*n*	Mo *K*α radiation, λ = 0.71073 Å
*a* = 5.8050 (3) Å	Cell parameters from 11538 reflections
*b* = 8.3566 (6) Å	θ = 1.0–35.0°
*c* = 8.7433 (6) Å	µ = 0.64 mm^−^^1^
β = 95.148 (4)°	*T* = 296 K
*V* = 422.43 (5) Å^3^	Prism, pale yellow
*Z* = 2	0.45 × 0.32 × 0.23 mm

### Bis(oxonium) ethane-1,2-disulfonate (I) Data collection

**Table:**

Bruker KappaCCD diffractometer	1848 independent reflections
Radiation source: fine-focus sealed tube	1768 reflections with *I* > 2σ(*I*)
Horizonally mounted graphite crystal monochromator	*R*_int_ = 0.075
Detector resolution: 9 pixels mm^-1^	θ_max_ = 34.9°, θ_min_ = 3.4°
CCD scans	*h* = −9→9
Absorption correction: integration Gaussian integration (Coppens, 1970)	*k* = −13→13
*T*_min_ = 0.748, *T*_max_ = 0.907	*l* = −14→14
17906 measured reflections	

### Bis(oxonium) ethane-1,2-disulfonate (I) Refinement

**Table:**

Refinement on *F*^2^	Secondary atom site location: difference Fourier map
Least-squares matrix: full	Hydrogen site location: difference Fourier map
*R*[*F*^2^ > 2σ(*F*^2^)] = 0.041	All H-atom parameters refined
*wR*(*F*^2^) = 0.121	*w* = 1/[σ^2^(*F*_o_^2^) + (0.0797*P*)^2^ + 0.1864*P*] where *P* = (*F*_o_^2^ + 2*F*_c_^2^)/3
*S* = 1.04	(Δ/σ)_max_ = 0.026
1848 reflections	Δρ_max_ = 0.62 e Å^−^^3^
76 parameters	Δρ_min_ = −0.93 e Å^−^^3^
3 restraints	Extinction correction: SHELXL-2014/7 (Sheldrick 2015b), Fc^*^=kFc[1+0.001xFc^2^λ^3^/sin(2θ)]^-1/4^
Primary atom site location: difference Fourier map	Extinction coefficient: 0.20 (2)

### Bis(oxonium) ethane-1,2-disulfonate (I) Special details

**Table:**

Geometry. All esds (except the esd in the dihedral angle between two l.s. planes) are estimated using the full covariance matrix. The cell esds are taken into account individually in the estimation of esds in distances, angles and torsion angles; correlations between esds in cell parameters are only used when they are defined by crystal symmetry. An approximate (isotropic) treatment of cell esds is used for estimating esds involving l.s. planes.

### Bis(oxonium) ethane-1,2-disulfonate (I) Fractional atomic coordinates and isotropic or equivalent isotropic displacement parameters (Å^2^)

**Table:**

	*x*	*y*	*z*	*U*_iso_*/*U*_eq_	
O1	0.03777 (17)	0.61584 (15)	0.29236 (14)	0.0319 (2)	
O2	0.4298 (2)	0.65073 (13)	0.21941 (13)	0.0302 (2)	
O3	0.30307 (19)	0.79554 (12)	0.43552 (13)	0.0308 (2)	
S4	0.27802 (4)	0.64978 (3)	0.34467 (3)	0.01890 (13)	
C5	0.3753 (2)	0.48838 (15)	0.46678 (14)	0.0231 (2)	
H5A	0.270 (5)	0.476 (3)	0.541 (3)	0.043 (6)*	
H5B	0.360 (4)	0.393 (3)	0.404 (2)	0.025 (5)*	
O6	0.7708 (2)	0.90258 (15)	0.39286 (16)	0.0363 (3)	
H61	0.822 (6)	1.009 (3)	0.357 (4)	0.066 (9)*	
H62	0.588 (4)	0.889 (4)	0.383 (4)	0.067 (9)*	
H63	0.797 (4)	0.795 (3)	0.350 (3)	0.041 (7)*	

### Bis(oxonium) ethane-1,2-disulfonate (I) Atomic displacement parameters (Å^2^)

**Table:**

	*U*^11^	*U*^22^	*U*^33^	*U*^12^	*U*^13^	*U*^23^
O1	0.0219 (4)	0.0345 (5)	0.0373 (5)	−0.0002 (3)	−0.0079 (4)	0.0012 (4)
O2	0.0341 (5)	0.0339 (5)	0.0238 (4)	0.0083 (4)	0.0096 (4)	0.0055 (3)
O3	0.0333 (5)	0.0227 (4)	0.0362 (5)	0.0020 (3)	0.0025 (4)	−0.0081 (4)
S4	0.01863 (17)	0.01917 (17)	0.01865 (17)	0.00184 (7)	0.00025 (10)	0.00054 (7)
C5	0.0218 (4)	0.0231 (5)	0.0234 (5)	−0.0027 (3)	−0.0032 (3)	0.0061 (4)
O6	0.0307 (5)	0.0320 (5)	0.0452 (6)	−0.0022 (4)	−0.0012 (4)	0.0000 (5)

### Bis(oxonium) ethane-1,2-disulfonate (I) Geometric parameters (Å, º)

**Table:**

O1—S4	1.4561 (10)	C5—H5A	0.94 (3)
O2—S4	1.4658 (11)	C5—H5B	0.97 (2)
O3—S4	1.4544 (10)	O6—H61	1.00 (2)
S4—C5	1.7804 (11)	O6—H62	1.06 (2)
C5—C5^i^	1.523 (2)	O6—H63	0.99 (2)
			
O3—S4—O1	112.37 (7)	S4—C5—H5A	108.0 (16)
O3—S4—O2	111.48 (7)	C5^i^—C5—H5B	110.7 (12)
O1—S4—O2	112.91 (7)	S4—C5—H5B	106.3 (12)
O3—S4—C5	107.23 (6)	H5A—C5—H5B	105 (2)
O1—S4—C5	106.51 (6)	H61—O6—H62	113 (3)
O2—S4—C5	105.82 (6)	H61—O6—H63	129 (2)
C5^i^—C5—S4	111.82 (11)	H62—O6—H63	93 (2)
C5^i^—C5—H5A	114.1 (17)		
			
O3—S4—C5—C5^i^	−57.98 (14)	O2—S4—C5—C5^i^	61.12 (14)
O1—S4—C5—C5^i^	−178.48 (12)		

Symmetry code: (i) −*x*+1, −*y*+1, −*z*+1.

### Bis(oxonium) ethane-1,2-disulfonate (I) Hydrogen-bond geometry (Å, º)

**Table:**

*D*—H···*A*	*D*—H	H···*A*	*D*···*A*	*D*—H···*A*
O6—H63···O2	0.99 (2)	2.62 (2)	3.1795 (17)	116 (2)
O6—H61···O2^ii^	1.00 (2)	2.02 (3)	2.9312 (16)	150 (3)
O6—H62···O3	1.06 (2)	1.92 (3)	2.9141 (16)	154 (3)
O6—H61···O3^iii^	1.00 (2)	2.60 (3)	2.9857 (16)	103 (2)
O6—H63···O1^iv^	0.99 (2)	2.14 (2)	3.0266 (18)	148 (2)

Symmetry codes: (ii) −*x*+3/2, *y*+1/2, −*z*+1/2; (iii) −*x*+1, −*y*+2, −*z*+1; (iv) *x*+1, *y*, *z*.

### Bis(oxonium) ethane-1,2-disulfonate (II) Crystal data

**Table:**

2H_3_O^+^·C_2_H_4_O_6_S_2_^2^^−^	*Z* = 1
*M**_r_* = 226.22	*F*(000) = 118
Triclinic, *P*1	*D*_x_ = 1.597 Mg m^−^^3^
*a* = 5.0371 (3) Å	Mo *K*α radiation, λ = 0.71073 Å
*b* = 5.5424 (2) Å	Cell parameters from 4728 reflections
*c* = 8.8188 (4) Å	θ = 1.0–32.6°
α = 98.426 (5)°	µ = 0.57 mm^−^^1^
β = 104.511 (3)°	*T* = 296 K
γ = 91.663 (4)°	Columnar, colorless
*V* = 235.22 (2) Å^3^	0.30 × 0.12 × 0.11 mm

### Bis(oxonium) ethane-1,2-disulfonate (II) Data collection

**Table:**

Bruker KappaCCD diffractometer	1708 independent reflections
Radiation source: fine-focus sealed tube	1192 reflections with *I* > 2σ(*I*)
Horizonally mounted graphite crystal monochromator	*R*_int_ = 0.131
Detector resolution: 9 pixels mm^-1^	θ_max_ = 32.6°, θ_min_ = 2.4°
CCD scans	*h* = −7→6
Absorption correction: integration Gaussian integration (Coppens, 1970)	*k* = −8→8
*T*_min_ = 0.813, *T*_max_ = 0.947	*l* = −11→13
7504 measured reflections	

### Bis(oxonium) ethane-1,2-disulfonate (II) Refinement

**Table:**

Refinement on *F*^2^	Secondary atom site location: difference Fourier map
Least-squares matrix: full	Hydrogen site location: difference Fourier map
*R*[*F*^2^ > 2σ(*F*^2^)] = 0.058	All H-atom parameters refined
*wR*(*F*^2^) = 0.163	*w* = 1/[σ^2^(*F*_o_^2^) + (0.0869*P*)^2^ + 0.0186*P*] where *P* = (*F*_o_^2^ + 2*F*_c_^2^)/3
*S* = 1.04	(Δ/σ)_max_ = 0.016
1708 reflections	Δρ_max_ = 0.66 e Å^−^^3^
76 parameters	Δρ_min_ = −0.67 e Å^−^^3^
0 restraints	Extinction correction: SHELXL-2014/7 (Sheldrick 2015b), Fc^*^=kFc[1+0.001xFc^2^λ^3^/sin(2θ)]^-1/4^
Primary atom site location: difference Fourier map	Extinction coefficient: 0.19 (3)

### Bis(oxonium) ethane-1,2-disulfonate (II) Special details

**Table:**

Geometry. All esds (except the esd in the dihedral angle between two l.s. planes) are estimated using the full covariance matrix. The cell esds are taken into account individually in the estimation of esds in distances, angles and torsion angles; correlations between esds in cell parameters are only used when they are defined by crystal symmetry. An approximate (isotropic) treatment of cell esds is used for estimating esds involving l.s. planes.

### Bis(oxonium) ethane-1,2-disulfonate (II) Fractional atomic coordinates and isotropic or equivalent isotropic displacement parameters (Å^2^)

**Table:**

	*x*	*y*	*z*	*U*_iso_*/*U*_eq_	
O1	0.6030 (4)	0.9888 (3)	0.7642 (2)	0.0387 (4)	
O2	0.6622 (4)	0.5760 (3)	0.8252 (2)	0.0378 (4)	
O3	0.2119 (4)	0.6995 (4)	0.7086 (3)	0.0417 (5)	
S4	0.50463 (11)	0.73087 (9)	0.72171 (6)	0.0267 (2)	
C5	0.5533 (5)	0.6324 (4)	0.5302 (3)	0.0290 (5)	
H5A	0.473 (7)	0.750 (6)	0.459 (4)	0.043 (8)*	
H5B	0.737 (7)	0.654 (6)	0.526 (4)	0.054 (9)*	
O6	0.1360 (5)	0.2554 (4)	0.8443 (3)	0.0477 (5)	
H61	0.318 (10)	0.173 (8)	0.827 (5)	0.083 (13)*	
H62	0.113 (7)	0.415 (6)	0.798 (5)	0.049 (9)*	
H63	−0.056 (8)	0.173 (6)	0.822 (5)	0.057 (10)*	

### Bis(oxonium) ethane-1,2-disulfonate (II) Atomic displacement parameters (Å^2^)

**Table:**

	*U*^11^	*U*^22^	*U*^33^	*U*^12^	*U*^13^	*U*^23^
O1	0.0405 (11)	0.0264 (8)	0.0464 (10)	−0.0019 (7)	0.0115 (8)	−0.0030 (7)
O2	0.0425 (11)	0.0396 (9)	0.0301 (8)	0.0109 (8)	0.0059 (7)	0.0062 (7)
O3	0.0235 (9)	0.0476 (10)	0.0538 (11)	0.0009 (7)	0.0125 (8)	0.0028 (8)
S4	0.0229 (3)	0.0261 (3)	0.0297 (3)	0.0012 (2)	0.0060 (2)	0.0014 (2)
C5	0.0289 (12)	0.0297 (11)	0.0271 (10)	−0.0025 (9)	0.0059 (9)	0.0034 (8)
O6	0.0415 (13)	0.0454 (11)	0.0535 (12)	0.0046 (9)	0.0092 (10)	0.0046 (9)

### Bis(oxonium) ethane-1,2-disulfonate (II) Geometric parameters (Å, º)

**Table:**

O1—S4	1.4625 (17)	C5—H5A	0.99 (3)
O2—S4	1.4509 (18)	C5—H5B	0.94 (4)
O3—S4	1.4532 (19)	O6—H61	1.07 (4)
S4—C5	1.777 (2)	O6—H62	1.02 (4)
C5—C5^i^	1.519 (4)	O6—H63	1.02 (4)
			
O2—S4—O3	112.00 (12)	S4—C5—H5A	109.0 (18)
O2—S4—O1	113.45 (11)	C5^i^—C5—H5B	110 (2)
O3—S4—O1	111.31 (11)	S4—C5—H5B	113 (2)
O2—S4—C5	106.21 (10)	H5A—C5—H5B	98 (3)
O3—S4—C5	107.20 (12)	H61—O6—H62	111 (3)
O1—S4—C5	106.16 (11)	H61—O6—H63	127 (3)
C5^i^—C5—S4	111.0 (2)	H62—O6—H63	107 (3)
C5^i^—C5—H5A	114.8 (19)		
			
O2—S4—C5—C5^i^	61.3 (3)	O1—S4—C5—C5^i^	−177.6 (2)
O3—S4—C5—C5^i^	−58.6 (3)		

Symmetry code: (i) −*x*+1, −*y*+1, −*z*+1.

### Bis(oxonium) ethane-1,2-disulfonate (II) Hydrogen-bond geometry (Å, º)

**Table:**

*D*—H···*A*	*D*—H	H···*A*	*D*···*A*	*D*—H···*A*
O6—H61···O1^ii^	1.07 (4)	1.93 (4)	2.991 (3)	170 (4)
O6—H62···O2^iii^	1.02 (4)	2.52 (3)	3.002 (3)	108 (2)
O6—H62···O3	1.02 (4)	1.97 (4)	2.945 (3)	158 (3)
O6—H63···O1^iv^	1.02 (4)	1.89 (4)	2.899 (3)	173 (3)

Symmetry codes: (ii) *x*, *y*−1, *z*; (iii) *x*−1, *y*, *z*; (iv) *x*−1, *y*−1, *z*.
